# The Cortico-Cortical and Subcortical Circuits of the Human Brain Language Centers Including the Dual Limbic and Language Functioning Fiber Tracts

**DOI:** 10.3390/brainsci16020142

**Published:** 2026-01-28

**Authors:** Arash Kamali, Nithya P. Narayana, Anastasia Loiko, Anusha Gandhi, Paul E. Schulz, Nitin Tandon, Manish N. Shah, Vinodh A. Kumar, Larry A. Kramer, Jay-Jiguang Zhu, Haris Sair, Roy F. Riascos, Khader M. Hasan

**Affiliations:** 1 Department of Diagnostic and Interventional Imaging, UTHealth Houston, Houston, TX 77030, USAkhader.m.hasan@uth.tmc.edu (K.M.H.); 2 McGovern Medical School, Houston, TX 77030, USA; nithya.p.narayana@uth.tmc.edu; 3 Department of Neuroscience, Rice University, Houston, TX 77005, USA; 4 Department of Neurology, University of Texas, Houston, TX 77030, USA; paul.e.schulz@uth.tmc.edu; 5 The Vivian L. Smith Department of Neurosurgery, McGovern Medical School, UTHealth Houston, Houston, TX 77030, USA; 6 Departments of Pediatric Surgery and Neurosurgery, McGovern Medical School, Houston, TX 77030, USA; manish.n.shah@uth.tmc.edu; 7 Department of Neuroradiology, MD Anderson Cancer Center, The University of Texas, Houston, TX 77030, USA; 8 Russell H. Morgan Department of Diagnostic and Interventional Imaging, The Johns Hopkins School of Medicine, Baltimore, MD 21205, USA

**Keywords:** aphasia, brain circuit, dorsal stream, dual stream, dorsal limbic network, expressive, language, limbic, receptive, superior longitudinal fasciculus, tractography, ventral stream, ventral limbic network

## Abstract

**Background/Objectives**: In recent years, MRI-based diffusion-weighted tractography techniques have uncovered additional white matter pathways that have significant roles in language processing and production. In this review, we aim to outline the major language centers of the brain and major language pathways along with association tracts that serve dual roles in both the language and limbic systems. According to the current dual-stream model of language processing, the brain’s language network is organized into a dorsal stream, responsible for mapping sound to articulation, and a ventral stream, which maps sound to meaning. **Materials and Methods**: The literature cited in this manuscript was identified through targeted searches of the *PubMed* database. Priority was given to peer-reviewed human studies, including original neuroimaging, cadaveric validation, and intraoperative stimulation studies. Non-peer-reviewed sources and publications lacking clear anatomical or functional correlation to language pathways were excluded. **Results**: Advances in functional MRI and diffusion weighted imaging techniques have revealed a more interconnected network, expanding our understanding beyond the classical dual-stream model of language processing. The Kamali limbic model proposed distinct ventral and dorsal limbic networks. Notably, several fiber pathways within the ventral limbic network may subserve both language and limbic functions. The association tracts with dual limbic-language functions form a critical basis for understanding the pathophysiology of language disorders accompanied by cognitive and emotional comorbidities observed in dyslexia, speech apraxia, aphasia, autism spectrum disorder, schizophrenia and post-traumatic stress disorder. **Conclusions**: Visualizing the language center and interconnected dual language and limbic fiber tracts highlights the importance of integrating language, executive function, and emotion in developing disease models and designing effective, targeted treatments for patients.

## 1. Introduction

### 1.1. Dual-Stream Model of Language

The prevailing model of language processing, known as the dual-stream model, divides the brain’s language network into a dorsal stream—which maps sound to articulation (expressive language)—and a ventral stream, which maps sound to meaning (receptive language) [1,2,3].

### 1.2. Methodological Overview of Diffusion-Weighted Imaging and Tractography

Diffusion-weighted imaging (DWI) is an MRI-based technique that exploits the anisotropic diffusion of water molecules along axonal fibers to infer white matter architecture in vivo. In organized white matter tracts, water diffusion is directionally constrained by axonal membranes and myelin, allowing reconstruction of fiber orientations using diffusion tensor imaging and more advanced high-angular-resolution diffusion models. Tractography algorithms use these diffusion profiles to estimate long-range cortico-cortical and cortico-subcortical pathways, providing a structural framework for large-scale brain networks. Compared with functional MRI and electrophysiological techniques, DWI-based tractography uniquely enables visualization of anatomical connectivity underlying distributed language and limbic systems, though it does not directly measure neural activity. Limitations include sensitivity to crossing fibers, partial volume effects, and the indirect inference of axonal continuity; therefore, tractography findings are best interpreted in conjunction with convergent anatomical, functional, and clinical evidence.

### 1.3. Primate Studies in Neuroanatomy Research

While in vivo DWI-based tractography methods are quite advanced, their limitations such as sensitivity to crossing fibers requires anatomical validations via dissection studies. Non-human primates, such as the rhesus monkey, are often studied in dissecting neuroanatomical studies due to the numerous structural similarities to the human brain. Knowing the regions of the brain that are connected by certain white matter fiber tracts identified in dissection primate studies allows for more precise tract identification via DWI tractography in humans [4]. Combining anatomical studies of white matter fiber tracts with lesion/microstructural integrity studies that reveal language function helps identify the association tracts that are involved in the organization of language.

### 1.4. Establishing the Function of White Matter Fiber Tracts

There are multiple ways to establish functional relevance to a specific group of fiber tracts through converging evidence including but not limited to the following: (1) correlations between tract microstructural integrity and behavioral or cognitive impairment in psychiatric disorders or stroke patients, by way of group comparisons in patient populations versus controls [5,6,7,8,9], (2) lesion–symptom mapping [10,11], (3) intraoperative electrical stimulation [12,13,14,15] and (4) intraoperative disconnection studies [16], for instance, in brain tumor resections, often supplemented by fMRI co-activation patterns.

### 1.5. Materials and Methods

In this manuscript, we searched the *PubMed* database for articles in English without restrictions on publication date using the following keywords: “Language tract”, “language pathway”, “language circuit”, “ventral stream”, “dorsal stream”, “superior longitudinal fasciculus”, “middle longitudinal fasciculus”, “dual stream”, “limbic and language”, “cerebellum and language”, “cerebellum and limbic”, “ventral premotor area”, “inferior parietal lobule and language”, “superior parietal lobule and language”, “visual word form area and language”, “dorsolateral prefrontal cortex and language”, “supplementary motor area and language”, “Wernicke’s area”, and “Broca’s area”. Articles were selected based on their relevance to the established and emerging human language centers, cortico-cortical and cortico-subcortical white matter tracts, and evidence supporting dual involvement in language and limbic systems. Priority was given to peer-reviewed human studies, including original neuroimaging, cadaveric validation, and intraoperative stimulation studies. Non-peer-reviewed sources and publications lacking clear anatomical or functional correlation to language pathways were excluded.

## 2. Association Tracts with Dual Function in the Language and Limbic Systems

**Figure 1:**
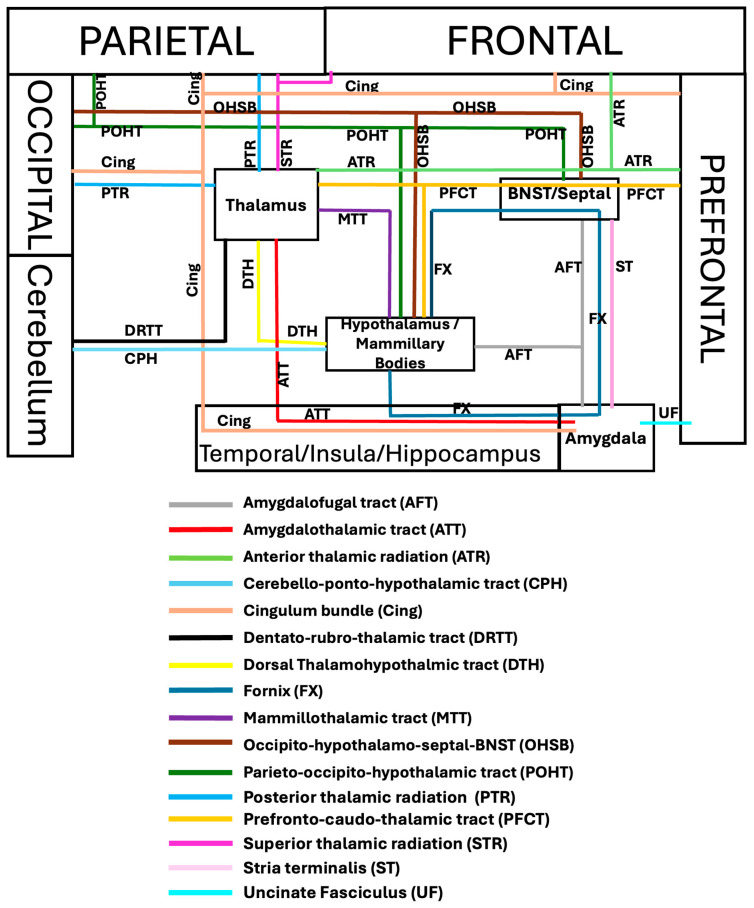
Schematic presentation of the Kamali ventral and dorsal limbic networks [14]. The ventral limbic network (emotions) includes connections between the central limbic gray matter nuclei (amygdala, hypothalamus, septal/BNST, hippocampus and thalamus) and the prefrontal, temporal, and insular cortices. The dorsal limbic network (cognition) links those nuclei to the posterior frontal, parietal, and occipital cortices, as well as to the cerebellum. A subset of fiber tracts is confined to the ventral limbic network such as the UF, PFCT, ATR. Certain fiber tracts are exclusive components of the dorsal limbic network including the DRTT, CPH, PTR, STR, POHT and OHSB. Other pathways are predominantly internuclear, linking gray matter nuclei to one another with zero or minimal direct cortical projections such as the ATT, MTT, AFT, ST, DTH and FX. The cingulum bundle (Cing) uniquely contributes to both the ventral and dorsal limbic networks by providing direct connections between the amygdala and all cerebral cortices.

**Figure 2:**
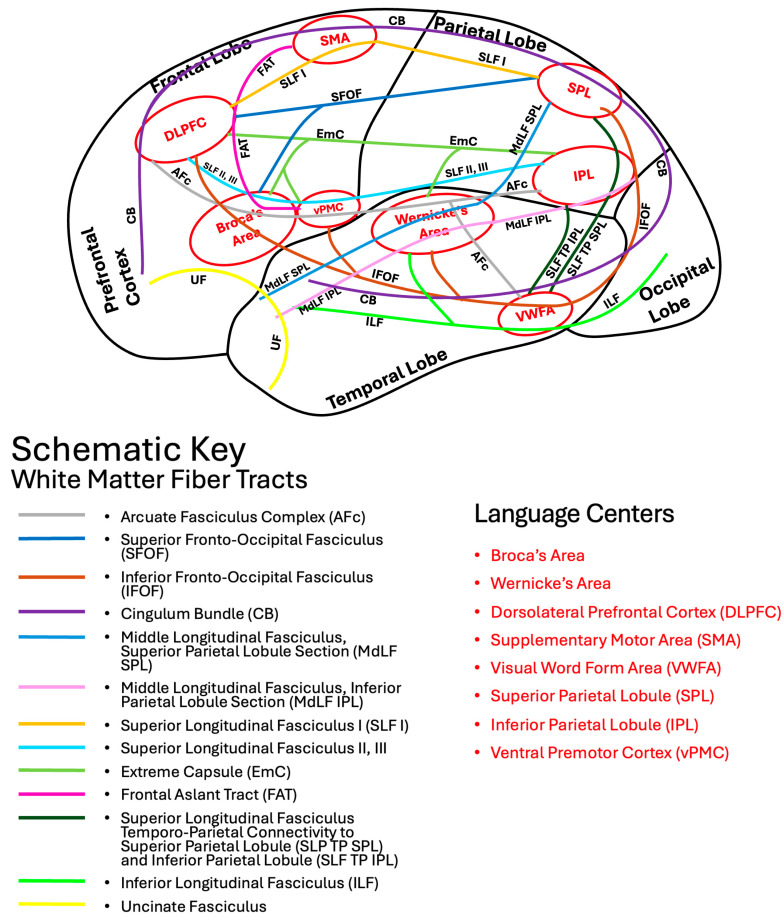
Schematic view of the white matter fiber tracts and language centers that compose the human brain language system.

There is significant overlap between the language and limbic systems in functioning and higher-level processing, including the integration of verbal and non-verbal memory and language production. The ventral and dorsal limbic circuits or networks were first introduced by Kamali et al., 2023 [17]. The ventral limbic network (emotions) comprises connections between the central limbic gray matter nuclei and the prefrontal, temporal, and insular cortices. In contrast, the dorsal limbic network (cognition) links those nuclei to the posterior frontal, parietal, and occipital cortices, as well as to the cerebellum (Figure 1) [17]. In contrast to limbic pathways, which mainly connect to at least one central gray matter nucleus, language pathways predominantly consist of cortico-cortical and cortico-subcortical connections and lack a central gray matter nuclear hub. Some limbic association fiber tracts have been demonstrated to contribute not only to limbic processing but also to language function. These dual function association tracts, described below in detail, include the uncinate fasciculus (UF), inferior longitudinal fasciculus (ILF), cingulum bundle (CB), inferior fronto-occipital fasciculus (IFOF), extreme capsule (EmC), prefrontal-caudo-thalamic tract (PFCT) or anterior thalamic radiations (ATR), and the arcuate fasciculus complex (AFc) (Figure 2).

### 2.1. The Uncinate Fasciculus

**Figure 3:**
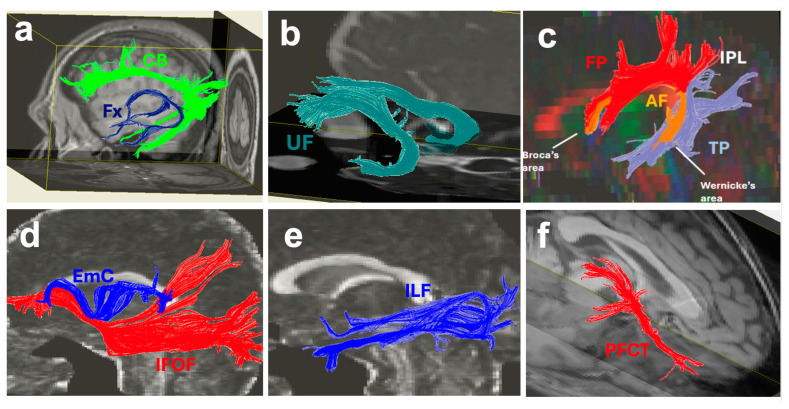
Three-dimensional reconstructions of the dual limbic–language functioning fiber tracts. (**a**) Lateral view of the cingulum bundle (CB) (green) (the CB connects the cingulate gyrus within the frontal and prefrontal regions directly with the parietal and occipital lobes and with the temporal lobe and amygdala) and fornix (Fx in blue, mostly a limbic tract). (**b**) Posterolateral view of the uncinate fasciculus (UF). (**c**) The AFc connects the insular cortex with the prefrontal cortex and temporal lobe. (**d**) The IFOF (red) connects the parietal and occipital cortices with the insular and prefrontal cortices. The EmC (blue) connects the insular cortex with the prefrontal cortex and inferior parietal lobule. (**e**) The ILF connects the amygdala and anterior temporal lobe with the Wernicke’s area and occipital cortex. (**f**) The PFCT connects the thalamus and the caudate nucleus to the prefrontal cortex.

The uncinate fasciculus (UF) is a key white matter fiber tract involved in both the limbic system and language processing areas of the brain. The UF is involved in the ventral stream of language by connecting the anterior temporal lobe (meaning) to the prefrontal cortex (executive function) [17,18]. The UF also connects to the amygdala and the insular cortex which are limbic structures (Figure 3b) [17]. The UF is involved in facilitating emotional and cognitive processing, allowing for higher-order functions in emotional regulation, memory, and language.

The UF connects the amygdala to the anterior insular cortex and to the orbitofrontal cortex (OFC) and the anterior cingulate cortex (ACC) in the prefrontal cortex which are limbic associated cortices, highlighting its involvement in the limbic system [17]. Through these connections, the UF facilitates emotional processing and social cognition. The UF’s connection to the hippocampus and the anterior insular cortex underscores its involvement in memory processes related to emotion, further exemplifying its involvement in the limbic system [17]. Studies showed involvement of the UF in emotional memories, recognition of fearful facial expressions, and cognitive situational reappraisal [8,19,20].

The structural integrity of the UF has been found to be disrupted in a number of psychiatric disorders. A meta-analysis [21] found that individuals with Major Depressive Disorder and bipolar disorder tend to have significantly lower reduced fractional anisotropy values in the UF compared to healthy controls, more strongly in the right than the left UF. Additionally, a meta-analysis [22] reported that individuals with bipolar disorder have significantly lower fractional anisotropy values in both the right and left UF and significantly higher radial diffusivity values in the right UF compared to healthy controls. This further supports the UF’s role in emotional regulation.

The UF connects the anterior temporal lobes and amygdala where multiple language pathways end (including the MdLF IPL, MdLF SPL, ILF) to the prefrontal cortex and orbitofrontal cortex where multiple other language pathways terminate at (including the IFOF, EmC, SLF II, III, and CB). These connectivities further support UF’s dual role in the limbic system and in language processing as implicated in the functions of semantic control and verbal episodic memory. The UF connects the frontal cortex to the temporal pole, which facilitates the retrieval and manipulation of semantic control for language production and comprehension. The CB creates a major limbic loop starting from the anterior prefrontal cortex to the amygdala in the mesial temporal lobes [23]. The UF specifically completes this loop by connecting the anterior temporal poles with the anterior prefrontal cortex (Figure 2). The UF also connects the anterior temporal poles where most of the ventral stream language pathways end (including the MdLF IPL, MdLF SPL, ILF) with the anterior prefrontal cortex where the CB ends [17]. By linking Wernicke’s area and the inferior parietal lobule (via the MdLF IPL and MdLF SPL) to the anterior cingulate gyrus—where the CB terminates—the UF closes the remaining loop in the language network and, with the support of the CB, incorporates the amygdala into the brain’s language circuits (Figure 2).

The UF facilitates the development of language-related memory functions and verbal episodic memory and the retrieval of word meaning from memory [24,25]. Furthermore, the integration of the limbic and language functions of the human brain via the UF is crucial to understanding the development of emotional nuances within language. The incorporation of emotion into language and communication is the basis for seamless social interactions. Nakajima et al. [10] provided an understanding of the UF’s involvement in the face-based mentalizing network, which is needed for recognizing and responding to social cues during communication. Due to these functions that connect sound to meaning, the UF is thought to be a part of the ventral stream [26].

### 2.2. The Extreme Capsule

**Figure 4:**
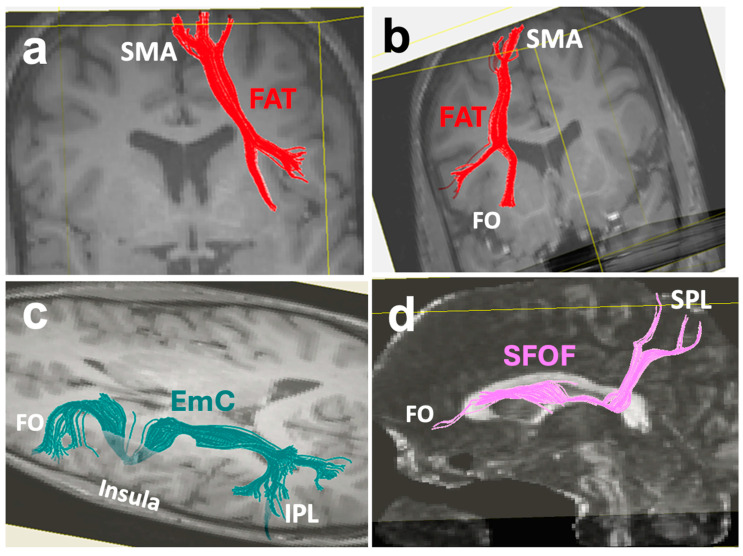
Three-dimensional reconstructions of the following fiber tracts: (**a**,**b**) frontal views of the bilateral frontal aslant tracts (FATs) connecting the supplementary motor area (SMA) with the dorsolateral prefrontal cortex and frontal operculum (FO); (**c**) the extreme capsule (EmC) connecting the inferior parietal lobule (IPL) with the insular cortex (Insula) and the frontal operculum (FO); (**d**) the superior fronto-occipital fasciculus (SFOF) connecting the superior parietal lobule (SPL) with the frontal operculum (FO) and prefrontal cortex.

The extreme capsule (EmC) is a rostro-caudal fiber bundle that is located between the claustrum and insular cortex, medial to the MdLF (Figure 3d and Figure 4c) [18,27,28,29]. The EmC is a major language pathway and part of the ventral stream of language in the human brain [18]. The EmC connects the language centers (Broca’s and Wernicke’s areas and IPL) with the prefrontal cortex and insular cortex [18]. Given the extensive insular cortex connectivity of the EmC, it also has a role as a limbic pathway in the human brain [17]. In rhesus monkeys, EmC fibers originate in the superior temporal sulcus and then split into the superior and inferior ramus rostrally. The superior ramus is situated within the white matter of the inferior frontal gyrus and projects to the vPMC, whereas the fibers of the inferior ramus run beneath the claustrum and terminate in the caudal part of the orbital frontal cortex, which correlates with the pars triangularis in humans [28]. A study using diffusion weighted tractography separates the EmC into three branches: the frontal, temporal, and parietal branches. The frontal branch connects the inferior frontal gyrus and orbitofrontal cortex with Broca’s area, the temporal branch connects the EmC with the middle part of the superior temporal gyrus, where Wernicke’s area is located, and the parietal branch connects to the angular gyrus in the IPL [29].

### 2.3. The Inferior Longitudinal Fasciculus

**Figure 5:**
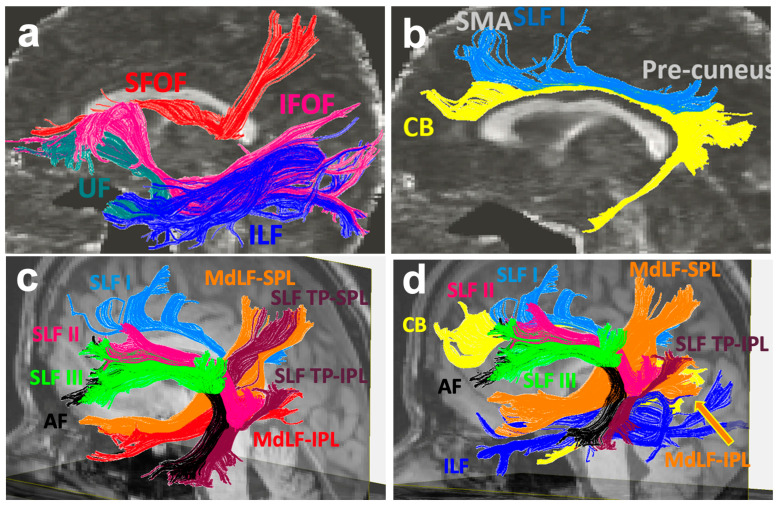
Three-dimensional reconstructions of the superior fronto-occipital fasciculus (SFOF), inferior fronto-occipital fasciculus (IFOF), uncinate fasciculus (UF), superior longitudinal fasciculi (SLF), arcuate fasciculus (AF), middle longitudinal fasciculi (MdLF), and cingulum bundle (CB). (**a**) The SFOF (red), IFOF (pink), UF (green), and ILF (dark blue) are shown. (**b**) The relationship between the SLF I (blue) and CB (yellow) is shown, with the SLF I connecting the supplementary motor area (SMA) with the pre-cuneus region. (**c**) The SLF I (light blue), SLF II (pink), SLF III (green), AF (black), MdLF SPL (orange), MdLF IPL (red), SLF TP SPL (purple), SLF TP IPL (purple) and the AF (black) are depicted. (**d**) The tractography of the SLF I (blue), SLF II (pink), SLF III (green), Cingulum bundle (CB), ILF (blue), MdLF SPL and MdLF IPL (orange), SLF TP IPL (purple) and AF (black) are illustrated.

The inferior longitudinal fasciculus (ILF) is a white matter tract of the human brain that has dual functioning in both the limbic system and as a part of the ventral stream of language processing. The ILF extends from the occipital lobe to the anterior temporal lobe (Figure 3e and Figure 5a,d) to allow for the integration of visual information with emotional, memory, and language functioning. In terms of functional anatomy, the ILF exhibits hemispheric asymmetry, with the right ILF being more involved with visual and emotional processing [30]; in contrast, the left ILF is more associated with the semantic retrieval and reading aspects of language processing [31]. The ILF’s dual role highlights its necessity in cognition and emotion as well as in linguistic functions.

The ILF connects the visual cortex with the visual word form area (VWFA) language center at the temporo-occipital junction. The ILF continues anteriorly toward the amygdala and fusiform gyrus in the anterior temporal lobe where it terminates [17]. The ILF is shown to facilitate functions of social interaction and emotional regulation, such as facial recognition and the processing of emotional stimuli, emphasizing its involvement in the limbic system. The ILF’s connections to the amygdala and fusiform gyrus enhance the ability to attune to social cues by supporting the recognition of emotionally charged visual information such as facial expression. Damage to the ILF has been linked to a decline in the ability to recognize facial emotions in Parkinson’s disease, highlighting its function in socio-emotional processing [32].

The ILF’s role in the limbic system is also displayed with its contributions to the ventral stream [26] through its functions of memory tied to emotional processing. By facilitating the transmission of visual and emotional information between the amygdala and hippocampus, the ILF integrates visual stimuli with emotional response [33]. Additionally, the ILF is involved in recognizing social cues and the ability to decode facial expressions. Damage to the ILF has been correlated to deficits in emotional empathy within autism spectrum disorder (ASD), further emphasizing its involvement in the limbic system [34].

The ILF’s involvement in language is evident in its function of visual word recognition and reading. For example, one investigation [35] showed that the left ILF is crucial for orthographic processing, which is a stage in the recognition of written words and their corresponding sounds. Furthermore, the ILF is integral to the visual processing pathway in language functions, as damage to the ILF can lead to impairments in reading comprehension and object recognition [36,37]. Through its connectivity between the anterior temporal lobe and occipital lobe, the ILF supports the integration of language with visual information to allow for reading and language comprehension tasks.

In terms of its dual roles in language and emotional processing, the ILF is involved in memory functions related to the limbic system. By connecting the occipital lobe and anterior temporal lobe to the hippocampus, the ILF links visual perception with memory storage and has an essential role in the consolidation and retrieval of visual memories [38]. Visual memory allows individuals to retain and recall visual information such as objects, scenes, or faces. For example, recognizing a familiar face involves visual recognition processed by the fusiform gyrus, and the recollection of emotions and previous interactions associated with that person, as supported by the hippocampus. Damage to the ILF can impede the integration of visual stimuli with emotional memories, leading to cognitive impairments such as prosopagnosia (inability to recognize faces) and object recognition. Furthermore, the ILF is involved in the processing of dynamic visual stimuli, such as recognizing changes in the environment, which requires the integration of past and present visual stimuli. As a result, damage to the ILF can also lead to difficulties in adapting to new environments and in using changing visual cues for other cognitive functions [39].

The ILF’s involvement in the limbic system and in language processing highlights its importance in the integration of visual, emotional, and cognitive information. Its connections between the occipital lobe, anterior temporal lobe, hippocampus, and amygdala allow it to support the dual roles of emotional processing and language functions.

### 2.4. The Cingulum Bundle

The cingulum bundle (CB) is a significant white matter tract of the brain that is involved in both language processing and the limbic system (Figure 3a and Figure 5b,d). Its connections between the prefrontal cortex, cingulate cortex, frontal, parietal and occipital cortices and limbic system structures of the temporal lobe such as the hippocampus and amygdala support a variety of functions related to executive function, visuosensory aspects, memory formation, and emotional responses [23]. Its connections between the prefrontal cortex and medial temporal lobe allow for memory regulation and emotional processing. The CB connectivity with the parieto-occipital cortices allows for visuosensory information processing while its connections with the hippocampus and amygdala allow for the consolidation of emotionally significant episodic memories [40,41].

The CB connects multiple language centers including the DLPFC, SMA, and SPL to one another (Figure 2) and also to the amygdala and hippocampus as part of the limbic system. By facilitating the connections with the medial frontal cortex, the CB supports cognitive flexibility in language tasks. Furthermore, the CB facilitates the integration of language comprehension with semantic memory to support higher-order linguistic tasks [42]. Various studies have shown that damage to the CB can lead to impairments in language functions such as verbal memory, comprehension, and sentence construction [43,44].

Furthermore, the CB is heavily implicated in declarative and procedural memory processing of language development. In terms of language development, declarative memory allows the storage of facts in vocabulary and grammar, while procedural memory allows the learning of rules in language. Damage to the CB can affect these memory systems, as shown in individuals with developmental language disorders who have difficulties in language acquisition and use [42,45].

The CB facilitates the connection between the prefrontal cortex and the hippocampus within the medial temporal lobe, allowing for memory formation and emotional processing. The prefrontal cortex is involved in memory-related executive functioning such as working memory, decision-making, and planning, and the hippocampus is necessary for long-term memory formation and the emotional encoding of memories. The CB supports the integration of emotions into memories to allow individuals to evaluate prior memories and make decisions based on the emotional context of the situation [45]. Damage to CB integrity in this connection has been linked to difficulties with emotional memory processing and emotional dysregulation, such as in schizophrenia, post-traumatic stress disorder (PTSD), obsessive–compulsive disorder (OCD), and autism spectrum disorder [40].

By connecting the cingulate cortex to the hippocampus, the CB allows for the coordination of emotional and memory processing within the limbic system. Within the limbic system, the ACC is involved in emotional regulation and decision-making, allowing for the modulation of emotional reactions. Emotional dysregulation such as anxiety and depression can occur due to disruption of the CB in this connection because of the ACC’s involvement in emotional responses. Furthermore, the CB’s link between the prefrontal cortex and hippocampus and amygdala allows the prefrontal cortex to integrate memories with emotional context into decision-making and to generate appropriate social responses [43,44,46].

### 2.5. The Inferior Fronto-Occipital Fasciculus

**Figure 6:**
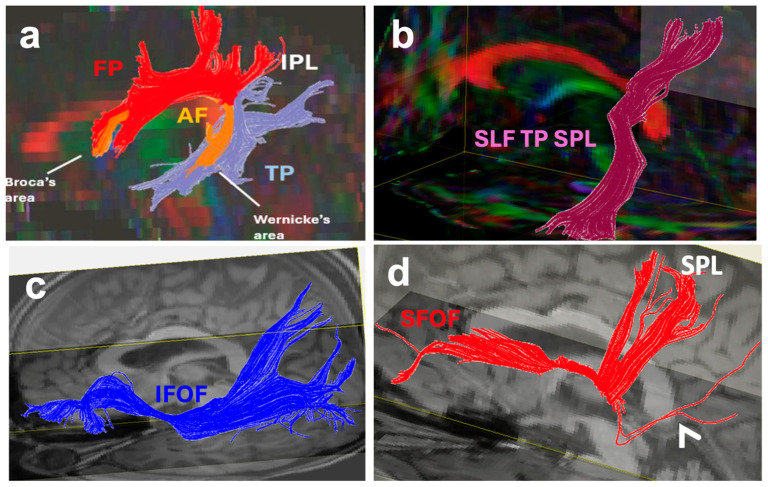
(**a**) Three-dimensional reconstructions of the arcuate fasciculus complex (AFc) in the background of diffusion weighted color-coded map, (**b**) superior longitudinal fasciculus temporo-parietal with superior parietal lobule connectivity (SLF TP SPL) in the background of diffusion weighted color-coded map, (**c**) the inferior fronto-occipital fasciculus (IFOF) and (**d**) the superior fronto-occipital fasciculus (SFOF). (**a**) The AFc consists of fronto-parietal connectivity from the inferior frontal gyrus (Broca’s area) to the IPL (red), temporo-parietal connectivity (Wernicke’s area) to the IPL (gray), and fronto-temporal connectivity (Broca’s area to Wernicke’s area) (orange). (**b**) The SLF TP SPL connects the posterior temporal lobe to the superior parietal lobule. (**c**) The IFOF connects the parietal and occipital lobes to the temporal, prefrontal and orbitofrontal cortices. (**d**) The SFOF connects the superior parietal (SPL) and occipital (arrowhead) cortices to the prefrontal and orbitofrontal cortical areas.

The inferior fronto-occipital fasciculus (IFOF) is a white matter tract that has a significant role in language processing and the limbic system (Figure 3d and Figure 6c). It connects multiple language centers within the frontal, parietal, and temporal lobes including the SPL, IPL, VWFA, Wernicke’s area, Broca’s area, vPMC, and DLPFC to the occipital lobe (Figure 2) to facilitate the integration of semantic information, visual processing, emotional regulation, and language. Both high angular diffusion spectrum imaging and cadaver dissection studies have shown the IFOF to originate in the OFC and frontal polar cortex and that it may have connectivity with the superior frontal gyrus and middle frontal gyrus [47,48]. The IFOF then connects the inferior frontal gyrus to the parietal and occipital cortices [47,48]. The extensivity of the IFOF allows interactions between linguistic and emotional functions within the brain [49]. Similarly to the superior fronto-occipital fasciculus (SFOF), which is connected to both the parietal and occipital lobes, the IFOF also connects to the superior parietal lobule, likely playing a role in transmitting visuospatial and sensory information for language processing [18,50].

The IFOF is a part of the ventral stream of language processing. The IFOF facilitates semantic processing within language functions by connecting the inferior frontal gyrus, where Broca’s area is located, SPL, VWFA, and the temporal lobe where Wernicke’s area is located with the occipital lobe. Through this connection, the IFOF allows for the transfer of visual and sensory information, such as written words processed by the occipital lobe, to linguistic information [11]. Damage to this connection causes semantic aphasia, which involves semantic processing deficits such as complications in producing coherent language and understanding the meaning of words [51]. One study showed that the IFOF’s connection of the frontal lobe to the VWFA enabled the connection of language networks with visual information to support reading and object recognition [12].

Previous studies have demonstrated a direct connection between the occipital cortex and the amygdala via the CB and ILF [23,30,38,39]. The CB also connects the parietal cortex directly with the amygdala [23]. The IFOF connects the parietal and occipital lobes to the insular cortex and temporal, prefrontal and orbitofrontal cortices [47,52]. Given the extensive limbic functionality of the OFC and insular cortex, the IFOF is likely involved in limbic function. To support this, a prior study of dementia patients concluded that reduced fractional anisotropy of the IFOF and forceps minor is correlated with apathy, and reduced fractional anisotropy of the SLF is correlated with disinhibition, both of which are considered limbic-related deficits [53]. Additionally, reduced fractional anisotropy of the IFOF was found in dementia patients with cognitive impairments such as reduced episodic memory and semantic deficits [6], supporting the dual function of the IFOF in both the limbic and language systems. Prior studies have also documented significantly reduced fractional anisotropy of the IFOF in patients with Major Depressive Disorder [9,54,55,56] and OCD [57], further implicating the IFOF’s role in emotional regulation. By integrating executive functioning with emotional memory, the IFOF allows for the moderation of emotional responses, which is significant for decision-making and adaptive behavior. By connecting visual stimuli to past memories, the IFOF allows for the production of appropriate emotional responses [48]. In terms of social cognition and the regulation of emotions, the IFOF is involved in the recognition of emotionally charged stimuli such as facial expressions and visual scenes [32]. Damage to the IFOF can disrupt a person’s ability to recognize and interpret facial emotions within social interactions. This disruption is relevant to schizophrenia and autism spectrum disorder, where affected individuals have difficulties in recognizing emotions and regulating social behavior [34,58].

Furthermore, the right IFOF is involved in non-verbal semantic cognition and spatial processing. One study found that direct electrostimulation and inactivation of the right IFOF was associated with dysfunctions of spatial awareness and attention toward emotional stimuli [59]. The findings in this study align with the concept that the IFOF is involved in processing non-verbal social cues, a function significant to emotional cognition [32].

The IFOF is an important white matter fiber tract involved in connecting multiple language centers, visual processing regions, and the limbic system to allow for the connection of linguistic, visual, and emotional information to support higher-order cognition. By facilitating semantic processing, the recognition of emotions, and social cognitions, the IFOF regulates social human behaviors and contributes to the ventral language stream [26]. Social interactions are conducted through the interplay of language and emotion, and knowledge of the connectivity of the IFOF facilitates a greater understanding of language and limbic interactions. 

### 2.6. The Arcuate Fasciculus and Arcuate Fasciculus Complex

The arcuate fasciculus (AF) is a significant white matter association tract that has a major role in language processing and speech production, which are functions that are a part of the dorsal stream of language. The AF is accompanied by short fronto-parietal, fronto-temporal and temporo-parietal association pathways (Figure 3c and Figure 6a) which are all together called the AF complex (AFc). The frontoparietal components of the AFc run side by side with the SLF II and SLF III connecting the inferior parietal lobule (IPL) to the prefrontal cortex.

The AF originates from the caudal superior temporal sulcus and the adjacent posterior temporal lobe and arches around the caudal sylvian fissure [60]. It passes dorsally through the white matter of the IPL below the SLF and ultimately connects Broca’s area in the frontal lobe with Wernicke’s area in the posterior temporal lobe [5,60,61]. The frontoparietal projections associated with AFc connect the IPL to the inferior frontal gyrus, and the temporo-parietal projections connect the IPL to Wernicke’s area and the VWFA (Figure 3c and Figure 6a).

Given the extensive connectivity with the insular cortex and OFC which are limbic associated cortices, the AFc likely has a role in the limbic system [42]. The AFc connects the insular cortex with the inferior frontal gyrus and prefrontal cortex, inferior and middle temporal gyrus and inferior parietal lobule [16]. Newer studies suggest that in addition to the language system, the AFc may also have a dual function in the limbic system, specifically related to memory and language learning as well as emotional encoding and regulation of memories and language. Studies in patients with mild traumatic brain injury have shown that damage to the temporal subsegment of the AFc is associated with memory deficits while damage to the frontal subsegment of the AFc is associated with language difficulties [62]. Furthermore, word learning is a key component of language processing which also impacts emotional processing and memory. Word learning has been shown to correlate with the strength of functional connectivity between Broca’s and Wernicke’s areas, suggesting that word learning depends largely on the integrity of the AFc [63]. Studies in developing readers have also shown that AFc connectivity is a key component of integration of auditory and visual information such as phonemes and graphemes, which is critical for functions such as letter-sound integration and ultimately encoding these associations for long-term development of literacy [64].

The AFc has a role in self-regulating emotions and emotional regulation. One study in military veterans suggests that white matter abnormalities in the AFc have a role in anger and aggression in this population [7]. The AFc also has a role in the recognition and processing of facial emotions. Axonal stimulation mapping studies have shown that face-based mentalizing and the ability to infer complex emotions from human facial expressions depend on the inferior-fronto-occipital tract and the superior longitudinal and arcuate fasciculi [7]. Overall, these studies further support the theory that the AF has a dual function in association with the limbic system to compose the emotional and memory aspects of language and communication.

### 2.7. The Prefronto-Caudo-Thalamic Tract and Anterior Thalamic Radiations

The prefronto-caudo-thalamic tract (PFCT) was first traced and described by Kamali et al. in 2010 as a large projection bundle which connects the thalamus to the basal ganglia and prefrontal cortex [65]. The PFCT is composed of corticostriatal pathways between the putamen and caudate to the prefrontal cortex and also has connections to the nucleus accumbens, BNST, and septal nuclei [17,65]. The PFCT connects the dorsomedial nucleus and anterior thalamic nuclei to the basal ganglia and nucleus accumbens and projects forward toward the prefrontal cortex, traversing through the caudate nucleus (Figure 3f). However, the anterior thalamic radiations (ATRs) directly connect the anterior thalamus to the cingulate cortex and prefrontal cortex by traversing through the anterior limb of the internal capsule, and not coursing through the caudate nucleus [17,65,66]. By communicating with multiple limbic nuclei, the PFCT has a key role in both language processing and the limbic system. A recent paper further describes the role and connectivity of the PFCT specifically in an updated model of the Papez circuit of the limbic system [17]. Through the thalamo-cingulate connectivity between the thalamus and anterior cingulate gyrus and prefrontal cortex, the ATR has a major role in completing the Papez circuit. The ATR and PFCT also contribute to multiple limbic and language circuits along with the CB and UF, connecting the prefrontal cortex with the ACC, thalamus, amygdala, and other language centers of the frontotemporal lobes [17].

Connections between the thalamus and prefrontal cortex are critical for language production as well as executive functions necessary for speech and communication, including planning, attention, and problem solving. The connection between the SMA and the prefrontal cortex to the thalamus via the thalamic radiations and PFCT allows the prefronto-thalamic connectivity to have a role in language processing and production. Subcortical signals from the basal ganglia and cerebellum are passed to the SMA through the thalamus which are largely inhibitory and are thought to function in language and speech networks to interrupt errors in processing that might delay language production, ultimately contributing to speech fluency [67]. The cerebellum is directly connected to the thalamus and SMA via the dentato-rubro-thalamic tract. Additional studies have suggested that these cerebellar and basal ganglia outputs to the thalamus and then ultimately to the prefrontal cortex via the PFCT and ATR are also involved in the timing of synchronizing thought processing to motor speech output and incoming speech perception and processing [68]. Overall, the prefronto-thalamic connectivity coordinates and integrates information that is processed for verbal communication.

It has been well established that thalamic lesions, especially lesions in the anterior nuclei of the thalamus, are associated with thalamic aphasias [69]. Disruption of the anterior and inferior thalamic peduncles in one study was associated with semantic defects, specifically when connections to the frontal cortex were disrupted. This loss of function, particularly difficulty with lexical–semantic retrieval, was thought to be due to the loss of neural fibers through the anterior nucleus of the thalamus [69]. This supports the theory that the anterior thalamus maintains the flow of coherent speech and syntax through the PFCT and ATR. Additionally, studies have illustrated that damage to the SMA clinically results in aphasias such as conduction aphasias, further supporting the role of the thalamic radiations in language processing and production [70].

The PFCT also has a dual role given its role in the limbic system. This tract connects to limbic areas and contributes to both emotional regulation and the integration of the verbal and non-verbal information from working memory. The prefronto-thalamic connectivity is key for nuanced language processing tasks such as understanding tone and context and integrating emotional content into verbal communication. The anterior thalamic nuclei also have a critical role in emotion and memory pathways via connections with specific medial prefrontal cortices [71]. Connecting the thalamus to the prefrontal cortex allows for cortical regulation of higher-order tasks such as modulating emotional responses to language and prioritizing actions based on the associated emotional significance. Specifically, the anterior medial nucleus was found to be crucial in retrieving information from long-term memory and solving problems in working memory [71]. One study in patients undergoing deep brain stimulation for the treatment of refractory epilepsy or depression-related symptoms demonstrated that stimulating the anterior thalamic nucleus leads to improved attentional capture by emotional stimuli [72]. Furthermore, it is well established that the PFCT and ATR allow for semantic associations with words and memories [73,74,75]. One study demonstrated that the anterior thalamus in particular has a role in incongruent information processing such as sentences with positive semantic content but with angry intonation [75]. Similarly to the inhibitory signals passed to the SMA through the thalamus via the thalamic radiations for the motor aspect of speech fluency, one study showed that deactivation of the anterior thalamus also has a role in gating memory encoding [76]. By effectively limiting the encoding of irrelevant information, the hippocampus and memory circuits prioritize transferring key information to the prefrontal cortex, thus impacting memory encoding and ultimately language processing.

Overall, these mechanisms allow the PFCT and thalamic radiations to join linguistic functions with emotional and motivational context and then ultimately encode this emotional context along with memories into long-term memory.

## 3. Association Tracts with Language Function

This section provides a brief overview of the white matter fiber tracts that are involved in the human brain language pathways but may not be directly involved in the limbic system. The white matter fiber tracts with mostly language function include the following:: the superior longitudinal fasciculus (SLF), the middle longitudinal fasciculus (MdLF), the extreme capsule (EmC), the superior fronto-occipital fasciculus (SFOF), and the frontal aslant tract (FAT).

The superior longitudinal fasciculus (SLF) pathways have a major role in language, spatial processing, emotion detection, and personality [14,33,77,78]. They connect areas in the perisylvian region, including the frontal, parietal, and temporal lobes [79], and they are believed to contribute to the dorsal language stream [26]. The superior longitudinal fasciculus fibers connect with the cingulate and insular cortices, both of which are limbic-associated cortices, suggesting potential direct connectivity with the limbic system [17,18,33]. The five subcomponents of the SLF are the SLF I, SLF II, SLF III, the arcuate fasciculus complex (AFc), and the temporo-parietal SLF (SLF TP), as illustrated by diffusion weighted tractography imaging [18]. The anatomy of these tracts is described below. The AFc is also described in further detail below along with additional association tracts with dual function in the language and limbic pathways.

### 3.1. The Superior Longitudinal Fasciculus I (SLF I)

The superior longitudinal fasciculus I (SLF I) is medial to the SLF II and SLF III and is located next to the CB (Figure 5b–d) [18]. This pathway was first traced in the human brain using diffusion weighted tractography technique by Kamali et al., 2014 and subsequently validated by human cadaver exam by Komaitis et al., 2020 [18,80]. The SLF I originates at the SPL, follows the cingulate gyrus, and connects with the supplementary and premotor areas in the frontal lobe (Figure 5b–d) [18,27]. The connectivity of the SLF I has been found to be similar in the rhesus monkey, supporting the fact that the SLF I is a separate white matter fiber tract from the CB [81]. The SLF I may be involved in utilizing body part location in the initiation of movement [27]. Given the close anatomical relationship between SLF I and the cingulate gyrus, a limbic cortical region, this fiber tract may also have a limbic functional component [17].

### 3.2. The Superior Longitudinal Fasciculus II (SLF II)

The superior longitudinal fasciculus II (SLF II) originates at the angular gyrus in the inferior parietal lobe and ends at the middle frontal gyrus, connecting the IPL with the prefrontal cortex and DLPFC [18,77]. It is located lateral to the SLF I and lateral to the corona radiata. The SLF II courses alongside the SLF III and the AF in an anterior-posterior direction, connecting the supramarginal gyrus, postcentral gyrus, and precentral gyrus (Figure 5c,d) [18]. Multiple studies have shown potential rightward lateralization of the SLF II [18,78,82]. The right SLF II is larger in volume and may have more involvement in visuospatial attention [78]. One study in children with developmental dyslexia highlighted that dyslexic children were significantly more lateralized in the right hemisphere in the SLF II than control children, suggesting that this asymmetry could contribute to deficiencies in reading capacity and further supporting the involvement of the SLF II in language [83]. The SLF II fibers connect the insular cortex, a limbic-associated cortex, with the prefrontal cortex and the inferior parietal lobule. Therefore, SLF II may serve a limbic role in addition to its established involvement in language function [17,18,33].

### 3.3. The Superior Longitudinal Fasciculus III (SLF III)

The superior longitudinal fasciculus III (SLF III) is more lateral than the SLF II and SLF I and originates rostral to the SLF II [18]. It connects the supramarginal gyrus in the IPL to the vPMC, prefrontal cortex, precentral gyrus, and pars opercularis and courses alongside the AF and SLF II (Figure 5c,d) [18,77]. The SLF III also exhibits asymmetry in anatomy. The right SLF III (but not the left SLF) connects to the pars triangularis, suggesting that the right SLF III could be involved in spatial awareness [77]. Electrostimulation of the left SLF III induces articulatory disorders such as anarthria or dysarthria, highlighting the importance of the SLF III in speech production [14]. By connecting the insular cortex, a limbic associated cortex, with the prefrontal cortex and IPL, the SLF III may have a limbic function as well [17].

### 3.4. The Arcuate Fasciculus and Arcuate Fasciculus Complex (AF, AFc)

The AF has been regarded as one of the major language pathways since the classical model of language processing was initially proposed believed to be a part of the dorsal language stream [84]. The AFc has a major role in multiple aspects of speech and language processing, including naming, comprehension, repetition, fluency, and spontaneous speech [5]. Studies have shown that damage to the AF may produce conduction aphasia, further supporting the AF’s major role in language and communication [85,86,87].

### 3.5. The Superior Longitudinal Fasciculus Temporo-Parietal with Superior Parietal Lobule Connectivity (SLF TP SPL)

The temporo-parietal superior longitudinal fasciculus (SLF TP) is a craniocaudal tract that has two branches: the inferior parietal lobule connectivity (SLF TP IPL) and superior parietal lobule connectivity (SLF TP SPL). This pathway was first discovered and traced in the human brain by Kamali et al., 2014 using the diffusion-weighted tractography technique and was later validated by a human cadaver study by Monroy-Sosa et al., 2019 [61,88]. The SLF TP IPL connects the posterior aspect of the superior and middle temporal gyri with the IPL, where some of the fibers insert into the angular gyrus and some insert into the supramarginal gyrus. The SLF TP SPL, which was discovered with the diffusion-weighted imaging (DWI) tractography technique, courses alongside the AF and SLF TP IPL and connects the caudal and posterior aspect of the middle and inferior temporal gyrus with the SPL (Figure 5d and Figure 6b) [18,61]. In relation to other white matter fiber tracts, the SLF TP SPL is medial to the SLF II and lateral to the ILF (Figure 5d). The connections of the SLF TP tracts with the visual cortex, auditory cortex, and temporo-parietal region exemplify this tract’s role in speech production, highlighting its contributions to the language pathways [61].

### 3.6. The Middle Longitudinal Fasciculus with Inferior Parietal Lobule Connectivity (MdLF IPL)

The middle longitudinal fasciculus (MdLF) connects to the inferior parietal lobule (IPL), forming the MdLF IPL pathway [18]. The MdLF IPL is a white matter tract that connects the angular gyrus of the IPL with the superior temporal gyrus and thus has a major role in language and other higher cognitive functions in humans [18]. The MdLF IPL originates from the rostral superior temporal gyrus, traverses horizontally through the superior temporal gyrus white matter, travels caudally through the sagittal stratum and posterior corona radiata, and terminates in the angular gyrus in the caudal IPL (Figure 5c,d) [61,89]. Studies in patients with IPL gliomas demonstrate that the IPL has a major role in language function and language deficits, further highlighting the importance of the MdLF IPL in language production and processing [90]. The MdLF is hypothesized to have a role in both the dorsal and ventral streams [91].

### 3.7. The Middle Longitudinal Fasciculus with Superior Parietal Lobule Connectivity (MdLF SPL)

The MdLF also connects to the superior parietal lobule (SPL) via the MdLF SPL. This pathway was first traced in the human brain by Kamali et al., 2014 and Wang et al., 2013 using the diffusion-weighted tractography technique and was eventually validated by human cadaver exam [18,92,93]. Similarly to the MdLF IPL, the MdLF SPL also originates in the rostral superior temporal gyrus. However, the MdLF SPL traverses superior to the MdLF IPL and inserts ultimately at the SPL just above the insertion point of the MdLF IPL (Figure 5c,d) [61].

### 3.8. The Extreme Capsule (EmC)

The EmC’s connections between the major language centers (Broca’s area, Wernicke’s area, and the IPL) suggest that it has a major role in the language pathway in humans. Given that the EmC has extensive connectivity with the insular cortex, a limbic associated cortex, it also has a role as a limbic pathway in the human brain [17]. Therefore, the EmC may have a major role in both language and limbic systems in humans [28,29,91,94,95]. A functional magnetic resonance imaging (fMRI) study performed on adults who completed the Boston Naming Test, a picture-naming test, revealed clusters of activation in the left hemisphere superior temporal gyrus and the inferior frontal gyrus pars orbitalis, suggesting that the EmC is the major connection between these regions. This picture-naming study highlights the possibility of the EmC being involved in the ventral stream and having a role in the semantic retrieval of words [94]. Multiple studies have examined the role of tau deposition on 18-F-flortaupecir PET imaging and MRI and its relationship with primary progressive aphasia and semantic dementia [96,97,98,99]. Abnormal cortical thinning and abnormal tau metabolism have been seen in amyloid-negative patients with primary progressive agnosia [97]. Tau accumulation and node to node propagation have been seen in patients with nonfluent primary progressive aphasia and involvement of the AF and other white matter tracts further underscores the crucial role of these tracts in language and communication [96,98].

### 3.9. The Superior Fronto-Occipital Fasciculus (SFOF)

The superior fronto-occipital fasciculus (SFOF) is a compact bundle that connects the frontal and parietal lobes. Studies show that the SFOF originates at the inferior frontal gyrus from Broca’s area (Figure 4d, Figure 5a and Figure 6d), courses under the corpus callosum medial to the corona radiata and lateral to the lateral ventricles and, lateral to the fornix and stria terminalis, and terminates at the SPL and occipital cortex [50,100]. In relation to other tracts with dual function in the language and limbic systems, the SFOF is also located superior to the IFOF, below and lateral to the CB, and below and medial to the SLF II (Figure 5a) [100]. The existence of this fiber tract has been well-described in monkeys [52,101]. In terms of function, its connections to the DLPFC, Broca’s area, and SPL suggest that the SFOF is part of the language system.

### 3.10. The Frontal Aslant Tract (FAT)

**Figure 7:**
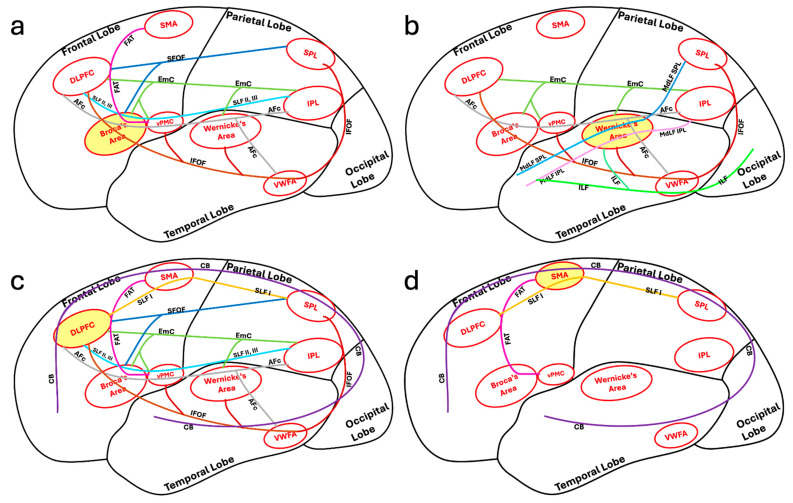
Schematic view of the language white matter fiber tracts through the following major language centers: (**a**) connections through Broca’s area; (**b**) connections through Wernicke’s area; (**c**) connections through the dorsolateral prefrontal cortex (DLPFC); (**d**) connections through the supplementary motor area (SMA).

The frontal aslant tract (FAT) is located in the frontal lobe, lateral to the corona radiata, connecting the SMA and pre-SMA with the posterior Broca’s area. It originates at the superior frontal gyrus (where the SMA is located), and from a coronal view, it travels obliquely to the pars opercularis of the inferior frontal gyrus (Broca’s area) (Figure 4a,b), hence the name “aslant” [102,103,104]. Some projections of the FAT extend to the dorsolateral prefrontal cortex (Figure 7c), the pars triangularis of the inferior frontal gyrus, and the inferior region of the precentral gyrus [102]. The FAT’s connections from the superior frontal gyrus to the inferior frontal gyrus have been found in both diffusion weighted and dissection studies [102,103,104], and the anatomy is comparable to those in monkeys [105]. The volume of the FAT exhibits a leftward asymmetry [102], which could reflect the leftward lateralization of language in most individuals.

The connections between SMA and Broca’s area, which are both major language centers, display that the left FAT is involved in the language pathway. Catani et al. [106] suggested that the FAT could be involved in verbal fluency, as microstructural abnormalities in the FAT were found in patients with agrammatic primary progressive aphasia. Another study using the Boston Naming Test by Jarret et al. [94] suggested that the FAT may be part of the dorsal stream when it comes to picture naming. Both studies hypothesized that the connections from the SMA to the pars opercularis could be involved in speech initiation [94,106].

Additionally, multiple direct electrical stimulation studies have shown that stimulating the FAT leads to the impairment of sentence production but not lexical retrieval [13,15], exemplifying that the FAT may be involved in higher-order language planning processes and not strictly motor processes. A study in patients with multiple sclerosis diagnosed with cognitive impairment also found that the bilateral FAT is associated with phonemic verbal fluency but not with semantic verbal fluency [104]. A case study published by Chernoff et al. [13] showed that the disruption of the left posterior aspect of the FAT led to issues with sentence production at the boundaries of grammatical phrases. Dragoy et al. [15] hypothesized that the left FAT supports spontaneous language fluency, and disruption of the terminations of the FAT (the SMA and Broca’s area) causes issues in speech planning and execution.

The right FAT may be involved in voluntary facial movements necessary for speech and emotion. A case study where damage to the right FAT and AF caused intraoperative Foix-Chavany-Marie syndrome, which is characterized by volitional palsy, revealed that the connections of the FAT were necessary for motor information from the SMA to communicate to the oropharyngeal cortex, highlighting the FAT’s role in voluntary facial movement [16].

## 4. Language Centers

The major language centers of the brain discussed in this review include Broca’s area, Wernicke’s area, the dorsolateral prefrontal cortex, the supplementary motor area or supplementary language area, the visual word form area, the superior parietal lobule, the inferior parietal lobule, the ventral premotor cortex, and the cerebellum.

### 4.1. Broca’s Area

Broca’s area is a crucial region of the brain associated with language production and processing. It is located in the anterior inferior frontal gyrus and encompasses Brodmann areas 44 and 45. The area was named after Paul Broca, a French physician and anatomist who linked damage to this region to deficits in speech production [107].

Regarding its connectivity, Broca’s area is linked to several regions of the brain. It is connected to the primary motor cortex via corticocortical fibers (Figure 7a), for example, to facilitate articulatory planning and execution. The primary motor cortex coordinates motor actions necessary for speech production [108]. Additionally, Broca’s area is connected to the DLPFC of the lateral prefrontal cortex and SMA of the medial frontal cortex through the FAT, which is an important white matter fiber tract connection for cognitive control aspects of speech production and fluency (Figure 7a) [109]. There is also direct connectivity of Broca’s area to the SPL and occipital lobe via the SFOF (Figure 7a) [79].

One of Broca’s area’s most significant connections is to Wernicke’s area, which is located in the posterior aspect of the superior temporal gyrus (Figure 7a). This connection is facilitated via the AF and AFc, which contains additional short pathways including the fronto-parietal and temporo-parietal connectivity (Figure 3c) [61] allowing for the bidirectional transfer of information for the comprehension and production of coherent speech [110]. The connection allows for the integration of linguistic and auditory information for language comprehension and production [111].

Broca’s area communicates with subcortical structures involved in the modulation and initiation of speech such as the basal ganglia and the thalamus via the PFCT which is an important limbic pathway for possible language function [17,65]. The PFCT is a cortical–subcortical syntax pathway that links Broca’s area to the striatum, an area of the basal ganglia, and thalamus to allow for syntactic processing and the production of grammatically complex sentences [112]. The thalamus is at the center of the limbic system’s connectivity. Thalamic infarcts have been documented to be a cause of thalamic aphasia [113,114,115,116]. The PFCT also connects the dorsomedial nucleus of the thalamus with the prefrontal cortex including the orbitofrontal cortex and frontal operculum which are limbic- and language-associated cortices. The direct connectivity of this pathway between Broca’s area, a primary language center, and the thalamus and orbitofrontal gyrus, which are involved in both limbic and language functions, emphasizes the dual limbic and language functionality of this pathway [117,118,119]. Broca’s area is also connected to the cerebellum and the cingulate gyrus, a limbic structure, via the fronto-ponto-cerebellar tract, further highlighting the limbic connectivity of Broca’s area [120].

Broca’s area is involved in the function of language processing, specifically the planning and execution of articulation. It aids in the construction and understanding of the grammatical structure of sentences within syntactic processing [121]. Additionally, it supports tasks requiring cognitive functions such as working memory and interference management during language production [122]. Broca’s area is essential for speech production, and damage can result in Broca’s aphasia, which is characterized by non-fluent speech, impaired syntax, and difficulty with speech production; however, comprehension remains intact [123].

Despite its crucial involvement in language production, Broca’s area is not the only major region involved in speech functions and outcomes. One study found that following a stroke, a network of regions is involved in the compensation and function of language production [123], indicating that long-term speech production was not solely dependent on Broca’s area.

Broca’s area is an integrative brain region essential for the production of language and syntactic processing. Its network of connections to areas of the cortex and subcortex highlights its important role in the different aspects of language function. Outlining the anatomy, connectivity, and function of Broca’s area showcases its importance in speech and language as well as how its damage can impact these language abilities.

### 4.2. Wernicke’s Area

Wernicke’s area is a region of the brain typically found in the left hemisphere necessary for language comprehension. It is located in the posterior part of the superior temporal gyrus, corresponding to Brodmann area 22. It was named after Carl Wernicke, who discovered it in the late 19th century. Wernicke’s area is associated with processing spoken and written language, and it is adjacent to the auditory cortex, emphasizing its role in processing auditory language information [124].

Wernicke’s area is situated at the left temporoparietal junction, which allows the region to integrate auditory information with cognitive functioning. Structural MRI scans and cortical thickness maps have expanded their boundaries to include parts of the middle temporal gyrus and the IPL [125]. Being involved in multiple interconnected regions, Wernicke’s area highlights the complexity of language comprehension.

Wernicke’s area has several primary pathways that connect it to other regions, including the AFc, which connects Wernicke’s area to Broca’s area in the frontal lobe (Figure 3c). This connection facilitates the transfer of processed auditory information for speech production and syntactic processing [126]. The AFc further connects the Wernicke’s area to the inferior parietal lobule (Figure 3c) [61]. Damage to the AF can lead to conduction aphasia, which is the inability to repeat words, although comprehension and speech production remain intact [127]. The AFc also connects Wernicke’s area with the insular cortex and the orbitofrontal gyrus, both of which are considered limbic-associated cortices [17,128]. The ILF also connects the Wernicke’s area with the amygdala which is a major limbic structure [129]. These connectivities indicate the dual limbic and language functionality of these fiber tracts.

In addition to the AFc and ILF, the MdLF connects the Wernicke’s area to the IPL (Figure 7b). The SLF along with the AFc link Wernicke’s area to the primary auditory cortex (Figure 7b), which may enable the direct processing of auditory stimuli for the quick interpretation and comprehension of spoken language [130]. Furthermore, the IFOF connects Wernicke’s area to the visual word form area and visual cortex (Figure 7b), allowing for the integration of visual and linguistic information essential for reading and writing [131]. In addition to the visual connectivity, the superior parietal connectivity of the IFOF and the MdLF also connect the Wernicke’s area directly to the SPL (Figure 7b) which could be involved in integration of the visual, attention, and spatial reasoning processing aspects of the language.

In terms of functionality, Wernicke’s area is involved in the processing of the phonological, lexical, and semantic aspects of language. The region enables the brain to comprehend words and sentences. Damage to Wernicke’s area can lead to Wernicke’s aphasia, which is characterized by fluent but nonsensical speech, severely impaired language comprehension, and difficulties in word retrieval [111].

The network of brain regions to support language comprehension also includes connections between Wernicke’s area, the angular gyrus, and the supramarginal gyrus via the AFc and part of the SLF, which support complex language functions such as reading and writing. The integration of these regions with Wernicke’s area further supports the perspective that language comprehension is a broad process involving multiple interconnected regions to support language production [132].

Wernicke’s area is situated in an area of the brain favorable for integrating auditory and linguistic information, making it crucial for language comprehension. Its extensive network of connections with regions such as Broca’s area, the auditory cortex, and visual cortex allows for the processing of spoken and written language (Figure 7b). Understanding Wernicke’s area’s anatomy and connectivity provides a deeper insight into its role in language function, and how damage to the area can impact language comprehension.

### 4.3. The Dorsolateral Prefrontal Cortex

The dorsolateral prefrontal cortex (DLPFC) is a key region of the frontal lobes, specifically located in the superior and middle frontal gyri, and includes Brodmann areas 9 and 46. The DLPFC is situated on the lateral surface of the frontal lobe and is anterior to the premotor and motor cortices and has one of the highest connectivity with the rest of the language system (Figure 2). It is bordered by the superior frontal sulcus and the inferior frontal sulcus. Its intricate structure and connectivity hint at its extensive involvement in higher-order cognitive processes [133].

The DLPFC may be linked with the VWFA and Broca’s area via the AFc (Figure 3b and Figure 7c). This link highlights the DLPFC’s role in language processing as well as the coordination of complex verbal functions. By connecting these regions, the AF enables the DLPFC to integrate phonological and semantic information necessary for fluent speech and reading [61,134,135].

Moreover, the DLPFC is connected to several other brain regions through multiple different white matter fiber tracts. For example, it is connected to the temporo-parietal cortical regions via the SLF, which allows for the integration of sensory and motor information and is crucial for advanced cognitive functioning such as working memory and executive control [130]. The DLPFC is also connected by the SLF to the posterior parietal cortex, a connection that supports the functions of spatial reasoning and attentional control [136]. Additionally, the DLPFC is linked to the ACC and the SMA via the CB (Figure 7c), which allows for various executive functions and motor planning [23,46]. Furthermore, the DLPFC is connected to the basal ganglia and the thalamus via the corticostriatal (such as the PFCT) and corticothalamic pathways (such as the ATR), which allows for decision-making and motor planning [17,65,137]. There is also direct connectivity of the DLPFC to the SPL via the SFOF and CB (Figure 7c) [138].

The DLPFC’s extensive network involvement is further shown by its connections to the occipital visual cortex via the CB and IFOF, which runs through the extreme capsule and temporal lobes and links the frontal lobes to the occipital and parietal lobes (Figure 7c), having a role in visual attention and processing complex visual stimuli [23,48,49]. Through its connections via the IFOF, the DLPFC is connected to the VWFA in the temporo-occipital junction and parietal lobe (Figure 7c) to support functions related to visual recognition and spatial reasoning.

The DLPFC is connected to components of the limbic system, particularly the amygdala and the hippocampus, through the UF and CB [23]. This white matter fiber tract link is crucial for emotional regulation, decision-making, and memory processing, which highlights the DLPFC’s role in integrating emotional and cognitive functions [17,136].

The DLPFC is central to the regulation and control of cognitive processes involved in executive functioning. It manages the manipulation of information in working memory, for example, which enables task planning, task switching, and inhibitory control. Its various connections with several brain regions allow it to be a center to integrate sensory inputs, regulate emotions, and coordinate complex behaviors, making it essential for goal-directed actions [137]. The FAT which is involved in cognitive control aspects of speech production and fluency also connects the DLPFC with the supplementary language area and Broca area (Figure 7c) [15,109].

Due to the extensive motor, sensory, limbic and cognitive connectivity, the DLPFC may be involved in various aspects of the pragmatic processing of the language including integration of prosody, discourse management, ambiguity resolution, interpretation of nonliteral meanings, inference making and error repair [67]. Damage to the DLPFC may result in psychiatric communication deficits rather than typical aphasic syndromes [67].

The DLPFC’s location in the brain and various connections support its role in executive functions and cognitive control. Its connections via the AF, SLF, CB, IFOF, and UF allow it to integrate information from the different senses, coordinate motor planning, regulate emotional responses, and support complex cognitive processing, highlighting its importance in higher-order thinking.

### 4.4. The Supplementary Motor Area (Supplementary Language Area)

The supplementary motor area (SMA) is crucial for the planning and coordination of movement. The SMA is located on the medial surface of the frontal lobe and is anterior to the primary motor cortex. The SMA is divided into the pre-SMA and SMA proper. The pre-SMA is anterior and is involved in higher-order motor planning such as the cognitive aspects of movement, and the SMA proper is posterior and is involved in the execution of motor actions [139].

The SMA is interconnected with several brain regions to form a complex network that supports motor control. It is connected to the primary motor cortex and premotor cortex to allow for the integration and execution of motor commands. It is also connected to the basal ganglia and thalamus to initiate and control movement. Furthermore, the SMA is connected to the prefrontal cortex via the superior thalamic radiations, cortico-thalamic, and cortico-striatal pathways [17], facilitating the link between motor planning with executive functioning and decision-making [103]. The SMA is also connected to the amygdala in the temporal lobe via the CB which likely facilitates the cognitive and emotional aspects of motor function [23].

The SMA is able to carry out its integrative functioning through its connections to various regions of the brain via white matter fiber tracts. For example, the SMA is connected to the primary motor cortex via corticocortical fibers, which are vital for coordinating motor actions by transmitting motor commands to the primary motor cortex for execution [103]. Additionally, the SMA is directly linked to the SPL via the SLF I and CB (Figure 6d). The SMA is indirectly linked to the language centers of the temporal lobe via the SLF TP SPL (Figure 7d) to facilitate the integration of sensory and motor information involved in coordinated movement and spatial awareness [61,130].

One significant connection of the SMA is to Broca’s area via the FAT (Figure 7d). This pathway enables the SMA to coordinate with Broca’s area, facilitating speech planning by supporting language production and articulation [15]. Additionally, the FAT links the SMA with the DLPFC (Figure 7d) to facilitate language production and speech fluency [13,15]. The integrity of the FAT is important for coordinating the complex motor actions required for verbal communication. Furthermore, the SMA is directly linked to the occipital visual cortex via the CB (Figure 7d) [23]. The SMA is shown to be involved in picture naming, suggesting its involvement in the integration of visual and motor functions required for this task [94].

In terms of functionality, the SMA is involved in the initiation of voluntary movements and the coordination of sequences of movements. Specifically, it is active during the preparation and planning phases of movement, before physical action is taken, to ensure the smooth and coordinated execution of complex motor tasks. Furthermore, the SMA is involved in the generation of self-initiated movements rather than those triggered by external stimuli, highlighting its function in actions that require internal motivation and self-control [139].

Additionally, the SMA’s involvement in speech and language production is indicative of its role in cognitive and motor integrated functions. Studies using repetitive transcranial magnetic stimulation have shown that disrupting the activity in the pre-SMA impairs speech production and non-speech oral motor gestures, indicating the importance of its complex motor control for effective speech [140].

Damage to the SMA may result in SMA syndrome which may present with contralateral hemiparesis, hemiapraxia, hemineglect, akinetic mutism, transient aphasia, diminished spontaneous speech and trouble with speech initiation [139,140].

The SMA is a multifaceted brain region essential for the planning, initiation, and coordination of motor actions. Its extensive network of connections with various cortical and subcortical areas signifies its key role in motor and cognitive functions related to speech and voluntary movement. Understanding the anatomy and connectivity of the SMA provides valuable information on its functionality regarding human behavior.

### 4.5. The Visual Word Form Area

The visual word form area (VWFA) is a key region of the brain that has an important role in the recognition and visual processing of written words. The VWFA is located in the left occipitotemporal cortex within the fusiform gyrus. It activates when individuals engage in reading or word-recognition tasks, which highlights its importance in the visual processing of language. Its location in the fusiform gyrus and involvement with the visual, linguistic, and attentional control networks highlight its importance in reading and word recognition. Additionally, its adaptability shown in the neuroanatomy of bilingual individuals signifies its importance in language processing.

**Figure 8:**
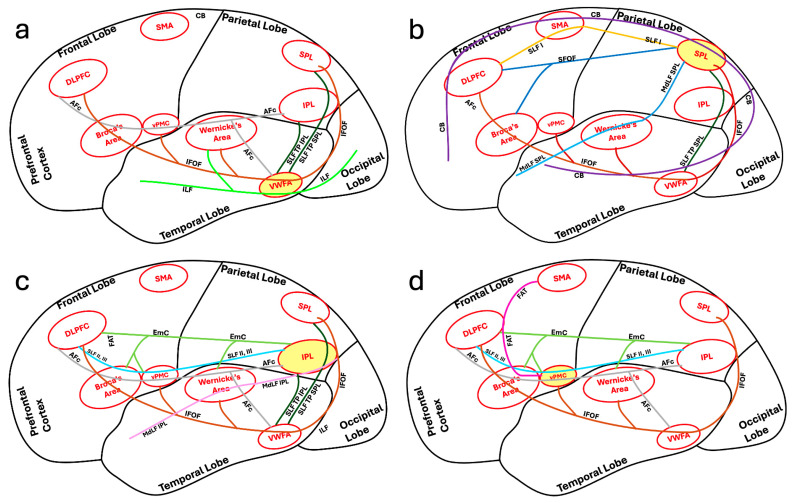
Schematic view of the language white matter fiber tracts through the following major language centers: (**a**) connections through the visual word form area (VWFA); (**b**) connections through the superior parietal lobule (SPL); (**c**) connections through the inferior parietal lobule (IPL); (**d**) connections through the ventral premotor cortex (vPMC).

The VWFA is connected to multiple brain regions that form a network to support its function in reading and language processing. One of its major connections is with the primary visual cortex via the ILF (Figure 8a) to receive visual input, which allows the VWFA to receive and process visual stimuli related to written words [134]. Additionally, the VWFA is connected to Broca’s and Wernicke’s areas via the IFOF (Figure 8a). These connections allow for the integration of visual word forms with linguistic information to facilitate language comprehension and production [64,141]. The VWFA is also directly connected to the SPL via the SLF TP SPL and superior parietal connectivity of the IFOF (Figure 8a), as described above, which further integrates the cognitive, attention and spatial processing aspects of language with this center [61].

Furthermore, the VWFA is a part of both the language and attentional control networks. It is connected to regions involved in attentional control such as the dorsal attention network, which allows for the selective focus on written words during reading tasks. The integration of attention with language processing ensures that visual attention is directed towards relevant stimuli, enhancing the efficiency of word recognition and reading [134]. The connection between the VWFA and the attention network highlights the interactions between visual processing, attention, and language necessary for efficient reading.

In bilingual individuals, the VWFA can show distinct patterns of activation and connectivity depending on the language that is being processed, suggesting that the VWFA is adaptable and can support the recognition of words in many different languages. The brain of a bilingual individual may show unique connectivity patterns in the VWFA, reflecting the adaptations that can form for managing multiple languages [142].

The most significant function of the VWFA is the rapid and efficient recognition of written words. It processes the orthographic features of words, which enables the brain to quickly identify and interpret them. This word-recognition processing is crucial for fluent reading and for linking visual input with phonological and semantic representations in the brain. The VWFA’s capacity in reading is further supported by its connectivity to regions involved in phonological processing and language comprehension via the AF [143].

Differences in the connectivity of the VWFA can have profound implications on reading abilities. Developmental dyslexia, for example, has been associated with changes in the white matter fiber tracts that connect the VWFA to other language regions of the brain. These alterations can negatively affect the efficient processing of written words, leading to difficulties in reading [83]. Therefore, understanding the connectivities of the VWFA can provide insights into the neuroanatomy that precipitates in reading disorders, and potential approaches for treatment intervention.

### 4.6. The Superior Parietal Lobule

The superior parietal lobule (SPL) is a fundamental component of the parietal lobe located on the dorsal side of the brain. It is located posterior to the primary somatosensory cortex and is bordered by the intraparietal sulcus inferiorly and parieto-occipital sulcus posteriorly [144]. The location of the SPL allows it to serve as a center for integrating sensory information and coordinating spatial perception and motor functions.

The SPL has extensive connectivity to other language centers of the brain through various white matter fiber tracts. One primary connection is through the SLF I, which links the SPL to the SMA and DLPFC, as described above (Figure 8b) [18,61]. The SPL is also directly connected to the VWFA and temporal lobe via the SLF TP SPL and parietal connectivity of the IFOF, as described above (Figure 8b) [61]. The SLF is subdivided into distinct components that facilitate communication between the SPL and different cortical areas, which support complex sensorimotor and cognitive functions [18,61]. For example, the SPL is connected to the DLPFC via the SLF I, SFOF and CB (Figure 8b) for attentional control and working memory processes. Additionally, the SPL is directly connected to the Wernicke’s area via the MdLF SPL, as described above (Figure 8b) [18,61,92,145]. Furthermore, the SPL is connected to the dorsal visual stream, also known as the “where” pathway, which extends from the occipital lobe and is essential for processing spatial aspects of visual information and guiding visuomotor actions [146].

The SPL’s role in sensorimotor integration is evident by its involvement in tasks requiring the perception and manipulation of objects in space. For example, the SPL engages in bimanual coordination, where it integrates sensory inputs from both hands to facilitate interactions with external objects. The SPL is primarily activated during these interactions, emphasizing its role in integrating tactile and proprioceptive inputs to guide coordinated movements [144].

The SPL’s involvement in somatosensory and motor functions is further exemplified in reaching tasks that involve depth perception. In reaching and guiding movements, hand position dominates over binocular eye position, emphasizing the necessity of the SPL in spatial awareness and motor control [147]. Furthermore, studies have shown that the SPL is crucial for updating the hand’s position in peripheral vision. This function becomes particularly evident in patients with optic ataxia, who struggle to reach for objects outside their central visual field due to impaired hand position updating [148].

Moreover, the SPL is connected to the prefrontal cortex via the SLF I (Figure 8b), a white matter fiber tract that supports executive functioning and decision-making processes related to spatial tasks [61]. Direct connectivity of the SPL is also shown with the occipital cortex and the amygdala in the temporal lobe via the CB (Figure 8b) [23]. These connections highlight the SPL’s role in higher-order cognitive functions, integrating visuosensory information with emotion and executive control to facilitate complex behaviors. Additionally, the SPL interacts with the angular gyrus and the superior temporal gyrus including Wernicke’s area to form a network that integrates auditory and language information; these connections allow for multimodal sensory processing and language comprehension [61,92,145].

In terms of functional connectivity, the SPL’s integration with the dorsal and ventral posterior cortex via associated white matter tracts such as the SLF I, CB and SFOF supports its role in coordinating sensory, motor and cognitive functions across different cortical regions [146]. These connectivities are crucial for controlling spatial orientation and executing complex motor actions, underscoring the SPL’s multifaceted input to sensorimotor integration and spatial cognition.

The SPL is a versatile brain region that has a crucial role in integrating sensory inputs, coordinating motor actions, and supporting higher-order cognitive functions. Its extensive network of connections with the frontal, temporal, and occipital lobes allows it to act as a center for sensorimotor and cognitive processing, highlighting its necessity for maintaining spatial awareness and executing coordinated movements. Outlining the anatomical and functional connectivity of the SPL provides valuable insights into its contributions to both basic sensory processing and complex cognitive functions.

### 4.7. The Inferior Parietal Lobule

The inferior parietal lobule (IPL) is a region of the brain located in the lateral aspect of the parietal cortex and has a significant role in various cognitive processes such as language, spatial reasoning, and attentional control. The IPL is inferior to the SPL and posterior to the postcentral gyrus. It is composed of the supramarginal gyrus and the angular gyrus and is bounded by the intraparietal sulcus. The two gyri are essential for integrating sensory information with higher cognitive functions [149].

The IPL has extensive connections with several brain regions via various white matter fiber tracts, which enable it to act as a focus for integrating sensory and cognitive information. One key tract is the superior longitudinal fasciculus (SLF II and SLF III respectively), which connects the IPL to the inferior frontal gyrus (including Broca’s area and the vPMC) as well as the DLPFC (Figure 8c) to support language processing and executive functioning [18,61]. The IPL is also connected to the prefrontal cortex and temporal lobe via the AFc (Figure 3c and Figure 8c), allowing it to support higher-order cognitive processes such as working memory and decision-making [92,150]. Additionally, the IPL is connected to the ventral visual stream via the ILF, which facilitates the integration of visual information with spatial and object recognition tasks [146].

The IPL is directly connected to the Wernicke’s area via the MdLF IPL (Figure 8c), as described above, which is involved in audiovisual integration and attention aspects of the language [18,92]. The IPL is also connected to the posterior temporal lobe near the temporo-occipital junction and VWFA via the temporo-parietal fibers of the IFOF and SLF TP IPL (Figure 5c,d and Figure 8c) [61]. This connectivity is with both the angular gyrus and supramarginal gyrus. Furthermore, the IPL is linked to Broca’s area in the frontal lobe and VWFA in the temporal lobe via the AFc (Figure 3c and Figure 8c), which is a complex of white matter fiber tracts critical for the IPL’s role in linguistic functions, as it facilitates language production and comprehension [97,105]. Another significant connection involves the EmC, which links the IPL to the inferior frontal gyrus to enable semantic processing and lexical retrieval [18,61]. The EmC connects the IPL to Broca’s area and the vPMC (Figure 8c), promoting language expression and enabling efficient communication and coordination between the two brain regions, which is necessary for complex language functions such as sentence construction and comprehension. Additionally, the EmC links the IPL to the DLPFC (Figure 8c) to facilitate the integration of sensory information with higher-order cognitive processes [29,61]. Tasks that involve the manipulation of and coordination of sensory and cognitive inputs, such as executive control, decision-making, and working memory, are supported through the EmC pathway [29].

The IPL is involved in a wide range of cognitive tasks. For example, it has a crucial role in the phonological and semantic aspects of language processing. Studies using fMRI and continuous theta burst stimulation have demonstrated the IPL’s active engagement in both phonological and semantic processing, which signifies its involvement in several different linguistic pathways [151]. Moreover, given the proximity of the IPL with the insular cortex and limbic system, the IPL could be involved in the functioning of working memory [15]. Functional connectivity studies have shown that variations in the connectivity of the IPL are linked to differences in working memory performance, which highlights its role in holding and manipulating information over short periods [152].

The IPL is heavily implicated in the coordination of motor actions, especially those requiring spatial awareness and attention, which highlights its integrative function in sensory and motor processes [153,154]. The IPL is able to integrate various sensory inputs from different modalities to construct a coherent spatial representation of the environment, further indicating its involvement with spatial attention and reasoning [152].

The IPL is a multifaceted brain region involved in the integration of various forms of sensory information to undertake the processing of higher cognitive functions. Its extensive connectivities with several different brain regions equips it to act as a hub for cognitive processing. Its role in language, working memory, spatial reasoning, and attention underscores its roles in both basic and cognitive processes [60,105].

### 4.8. The Ventral Premotor Cortex

The ventral premotor cortex (vPMC) is an area of the frontal lobe that has both motor and cognitive functions. The vPMC is located anterior to the primary motor cortex and inferior to the dorsal premotor area, in the inferior region of the premotor cortex. It is located at the inferior end of the precentral gyrus just next to Broca’s area, within the pars opercularis, an area of the brain involved in syntax in language production. The location of the vPMC allows it to integrate motor and cognitive information with sensory inputs to conduct a variety of functions [155]. Studies showed that the damage to the vPMC may cause the highest rate of speech arrest, even higher than Broca’s area [156,157]. The vPMC is connected to multiple different regions of the brain to support its role in the integration of motor planning and speech production. Through parieto-frontal tracts, it connects to the anterior intraparietal area of the IPL through the SLF III and EmC (Figure 8d) to allow for the integration of somatosensory inputs with motor planning [18,158]. The vPMC’s connection with the IPL allows for the transformation of object shape and orientation information into motor actions such as the manipulation of said objects. Furthermore, the vPMC’s connections with the superior temporal sulcus facilitate biological motion perception, which is the ability to easily perceive and recognize complex human movements. The ability of biological motion perception allows one to predict others’ actions and behaviors, which is necessary for seamless communication and socialization [159].

Parts of the inferior frontal cortex that contain the vPMC are also connected to the middle and inferior temporal gyri through the IFOF and AFc. Its connections through the IFOF to the VWFA (Figure 8d) allow for the integration of visual and linguistic information for the facilitation of object-naming and recognizing actions [102]. The AFc connects the vPMC and Broca’s area with Wernicke’s area (Figure 8d), allowing for the vPMC to contribute to motor planning in speech. By linking auditory input from Wernicke’s area with motor planning in speech through the vPMC and Broca’s area via the AFc, speech repetition and spoken language production can occur. Furthermore, the SLF links the vPMC with Broca’s area and the DLPFC (Figure 8d). The vPMC’s connection to Broca’s area through the SLF allows for functions of linguistic planning such as timing during articulation in speech. Additionally, the vPMC’s connection to the DLPFC allows for the integration of working memory and attentional control in speech, functions needed for sequential thought processes during conversation and verbal fluency. Lastly, the vPMC’s connection with the SMA via the FAT allows for the planning, initiation, and fluency of speech [160].

Mirror neurons located in the vPMC allow for the execution of planned actions from observing similar actions performed by others. This further exemplifies the vPMC’s use of biological motion perception in understanding and learning observed actions [161]. By processing the kinematics of actions and through imitation, the vPMC contributes to fine motor control and oral actions, such as hand and mouth movements [162].

The EmC is extensively connected to the insular cortex which is a known limbic cortex. Given the close anatomical proximity and connectivity of the vPMC with the insular cortex via the EmC, a dual limbic and language functionality of these structures is highly plausible. [28,29,91,94,95]. The close connectivity of the EmC and insular cortex with the vPMC, supports the integration of the motor language with the cognitive information and emotional context.

Through its various connections to the prefrontal, parietal, and temporal lobes, the vPMC is a center for motor understanding, planning, and execution as well as verbal communication. Through the integration of sensory information and motor commands, the vPMC is a crucial area for social cognition and is vital to action and language-based functions of the human brain.

### 4.9. The Cerebellum

The cerebellum does not generate language on its own but acts as a crucial modulator especially for the timing, fluency, and coordination of linguistic processes. Through its connections with multiple central limbic nuclei, the cerebellum has a dual role in modulating both language and limbic functions 31 [163]. The cerebellum is involved in articulation and speech production including the coordination of precise timing and sequencing of speech muscles [164]. The cerebellum is also involved in syntax and grammar processing, verbal working memory, and error monitoring and correction. The cerebellum connects to language areas via cortico-ponto-cerebellar and cerebello-thalamo-cortical loops [65,120,165]. Damage to parts of the cerebellum can cause “cerebellar cognitive affective syndrome”, which includes language deficits like agrammatism, dysarthria, and impaired verbal fluency [166,167].

The cerebellum is directly linked with the limbic structures such as the thalamus and the anterior limbic nuclei such as the hypothalamic nuclei via the cerebello-thalamic and cerebello-hypothalamic connectivity such as the dentato-rubro-thalamic tract and cerebello-ponto-hypothalamic tract [163,165]. The cerebellum is also indirectly connected with the orbitofrontal cortex (OFC) and the anterior cingulate cortex (ACC) which are limbic associated cortices involved in emotional control via the cerebello-thalamo-cortical loops [163]. Cerebellar infarcts are shown to be associated with aphasia and loss of speech fluency [168,169,170]. The cerebellum is also directly connected to wide areas of cerebral cortices including the prefrontal cortex and cingulate gyrus via the cortico-ponto-cerebellar pathways such as the fronto-ponto-cerebellar tract [120]. The fronto-ponto-cerebellar tracts are also connected to Broca’s area [120]. This further affirms the dual limbic and language functionality of the cerebellum.

## 5. Conclusions, Future Directions, and Clinical Applications

Knowledge of specific roles and connections of each language center has profound theoretical and clinical implications. Disorders such as dyslexia, speech apraxia, and aphasia can arise from disruptions to specific language centers and their connectivity, and knowledge of the specific tracts involved may guide treatment. Deeper insight into the complex, dual-functioning limbic- and language-associated fibers may offer a more unified understanding of how language, emotional balance, social cognition, and memory are seamlessly integrated. This information may aid in conditions such as schizophrenia and autism spectrum disorder presenting with both language and emotional dysfunction. Future research may further explore rehabilitation strategies that promote the recovery of these affected pathways through targeted stimulation.

## Data Availability

No new data were created or analyzed in this study.

## Abbreviations

The following abbreviations are used in this manuscript:

DWI	Diffusion weighted imaging
MRI	Magnetic resonance imaging
PET	Positron electron tomography
SLF I	Superior longitudinal fasciculus I
SLF II	Superior longitudinal fasciculus II
SLF III	Superior longitudinal fasciculus III
SLF TP SPL	Superior longitudinal fasciculus temporo-parietal connectivity to superior parietal lobule
MdLF IPL	Middle longitudinal fasciculus with inferior parietal lobule connectivity
MdLF SPL	Middle longitudinal fasciculus with superior parietal lobule connectivity
EmC	Extreme Capsule
SFOF	Superior fronto-occipital fasciculus
FAT	Frontal aslant tract
UF	Uncinate fasciculus
ILF	Inferior longitudinal fasciculus
CB	Cingulum bundle
IFOF	Inferior fronto-occipital fasciculus
PFCT	Prefronto-caudo-thalamic tract
ATR	Anterior thalamic radiations
BNST	Bed nucleus of the stria terminalis
AF	Arcuate fasciculus
AFc	Arcuate fasciculus complex
DLPFC	Dorsolateral prefrontal cortex
SMA	Supplementary motor area
VWFA	Visual word form area
SPL	Superior parietal lobule
IPL	Inferior parietal lobule
vPMC	Ventral premotor cortex
OFC	Orbitofrontal cortex
ACC	Anterior cingulate cortex
PTSD	Post-traumatic stress disorder
OCD	Obsessive compulsive disorder
ASD	Autism spectrum disorder
